# 2-O-Methylmagnolol upregulates the long non-coding RNA, GAS5, and enhances apoptosis in skin cancer cells

**DOI:** 10.1038/cddis.2017.66

**Published:** 2017-03-02

**Authors:** Tong-Hong Wang, Chieh-Wen Chan, Jia-You Fang, Ya-Min Shih, Yi-Wen Liu, Tzu-Chien V Wang, Chi-Yuan Chen

**Affiliations:** 1 Graduate Institute of Health Industry Technology and Research Center for Industry of Human Ecology, College of Human Ecology, Chang Gung University of Science and Technology, Kwei-Shan, Tao-Yuan 333, Taiwan; 2 Tissue Bank, Chang Gung Memorial Hospital, Kwei-Shan, Tao-Yuan 333, Taiwan; 3 Pharmaceutics Laboratory, Graduate Institute of Natural Products, Chang Gung University, Kwei-Shan, Tao-Yuan 333, Taiwan; 4 Department of Anesthesiology, Chang Gung Memorial Hospital, Kwei-Shan, Tao-Yuan 333, Taiwan; 5 Department of Molecular and Cellular Biology, College of Medicine, Chang Gung University, Kwei-Shan, Tao-Yuan 333, Taiwan

## Abstract

Magnolol, a hydroxylated biphenol compound isolated from the bark of *Magnolia officinalis*, has been shown to exhibit anti-proliferative effect in various cancer cells, including skin cancer cells. Methoxylation of magnolol appears to improve its anti-inflammatory activity, yet the effect of this modification on the agent's antitumor activity remains unknown. In this work, we report that 2-O-methylmagnolol (MM1) displays improved antitumor activity against skin cancer cells compared to magnolol both *in vitro* and *in vivo*. The increased antitumor activity of MM1 appears to correlate with its increased ability to induce apoptosis. DNA microarray and network pathway analyses suggest that MM1 affects certain key factors involved in regulating apoptosis and programmed cell death. Interestingly, the level of the long non-coding (lnc) RNA of *growth arrest-specific 5* (GAS5) was increased in MM1-treated cells, and inhibition of lncRNA GAS5 inhibited MM1-induced apoptosis. Conversely, overexpression of lncRNA GAS5 inhibited cell proliferation and promoted cell apoptosis in skin cancer cells. The expression of lncRNA GAS5 in the skin cancer tissues was found to be lower than that in the adjacent normal tissues in a majority of patients. Taken together, our findings suggest that MM1 has improved antitumor activity in skin cancer cells, and that this is due, at least in part, to the upregulation of lncRNA GAS5 and the enhancement of apoptosis.

Skin cancer represents a major and growing public health problem. The three most common forms of skin cancer are basal cell carcinoma, squamous cell carcinoma, and melanoma. Basal cell carcinoma and squamous cell carcinoma are together known as non-melanoma skin cancers and are both derived from keratinocytes, whereas melanomas are derived from melanocytes.^1^ Squamous cell carcinomas can undergo metastasis, basal cell carcinomas rarely show this progression, and melanomas can be highly metastatic.

Although surgical removal remains the primary treatment for skin cancer, herbal therapy is becoming increasingly popular among physicians and patients.^2^ *Magnolia officinalis* is widely used in traditional Chinese medicine because of its various pharmacological activities.^3, 4, 5^ Magnolia bark is rich in the extensively investigated biphenol compound, magnolol (5,5′-diallyi-2,2′-dihydroxybiphenyl, C_18_H_18_O_2_).^6^ The cytotoxic and anti-angiogenic activities of magnolol appear to reflect the presence of hydroxyl and allylic groups on a biphenolic moiety.^7^ An early study showed that magnolol had remarkable inhibitory effects on mouse skin tumor promotion in an *in vivo* carcinogenesis test.^8^ More recently, magnolol has been reported to exert antitumor activity by inhibiting proliferation and inducing apoptosis,^9, 10, 11, 12, 13^ countering metastasis,^14, 15, 16^ and suppressing angiogenesis.^17, 18^ Magnolol induces apoptosis in the cells of many human cancers, including gallbladder cancer, non-small cell lung cancer, prostate cancer, human breast cancer, bladder cancer, colon cancer, and skin cancer.^9, 10, 11, 12, 14, 18, 19, 20, 21, 22, 23, 24^ Therefore, magnolol has been suggested as a potential apoptosis-targeting drug.^25^ Together, the results from the existing *in vivo* and *in vitro* studies have indicated that magnolol is a promising candidate for the development of new strategies for preventing and/or treating skin cancers in humans.

Previous studies demonstrated the methoxylation of honokiol enhanced the anti-inflammatory effect.^26^ In an attempt to optimize the anti-inflammatory ability of magnolol, we previously synthesized two derivatives of magnolol bearing one or two methoxy moieties, and designated these agents ‘2-O-methylmagnolol' (MM1) and ‘dimethylmagnolol' (M2M), respectively.^27^ However, although we showed that MM1 and M2M exhibited improved anti-inflammatory activity, the cytotoxic mechanisms and *in vivo* anti-carcinogenic effects of these compounds remained largely unknown. In the present study, we compared the antitumor activity of magnolol, M2M and MM1, against skin cancer cells *in vitro* and *in vivo*, and explored the underlying cytotoxic mechanisms.

## Results

### Cytotoxic effects of magnolol, M2M and MM1 in skin cancer cells

**Figure 1:**
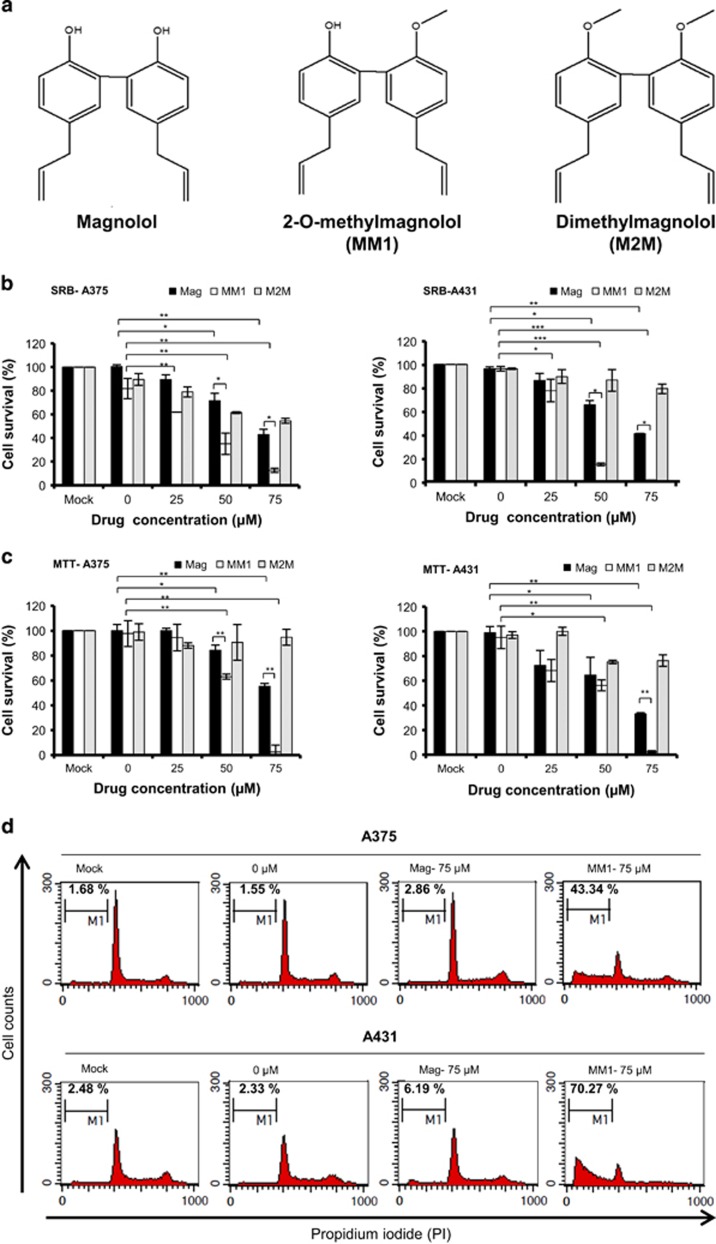
Structures and cytotoxic activities of magnolol, M2M, and MM1. (**a**) Chemical structures of magnolol, M2M, and MM1. (**b** and **c**) A375 and A431 skin cancer cell lines were treated with various concentrations of magnolol, M2M, or MM1 for 24 h. Their cytotoxic effects and the viabilities of the treated cells were detected with (**b**) SRB assays and (**c**) MTT assays. (**d**) Flow cytometric analysis of cell cycle. A375 and A431 cells were treated with 75 *μ*M of magnolol (Mag) or MM1 for 24 h. The treated cells were stained with propidium iodide and analyzed by flow cytometry. The percentages of cells in the sub-G1 region (M1) are indicated. Data shown in **b** and **c** are expressed as the mean±S.D. of two independent experiments. Symbols: *P<0.05; **P<0.01; and ***P<0.001, as analyzed by unpaired *t*-tests. Data shown in **d** are from one of two similar results

To optimize the anti-inflammatory ability of magnolol, we previously synthesized methoxylated derivatives of magnolol (Figure 1a). We found that whereas magnolol displayed a moderate cytotoxic effect against keratinocytes at 10 *μ*g/ml, M2M and MM1 displayed little or no such effect.^27^ Thus, this methoxylated compound appeared to have lower cytotoxicity against normal keratinocytes compared to magnolol. Here we set out to evaluate the antitumor effects of magnolol, M2M and MM1. We first examined the cytotoxic effects of these compounds in the skin cancer cell lines, A375 (melanoma) and A431 (squamous carcinoma). As shown in Figure 1b, MM1 displayed a greater cytotoxic effect against A375 and A431 cells compared to magnolol and M2M using sulforhodamine B (SRB) assay. Further examination showed that MM1 more significantly inhibited cell proliferation than magnolol and M2M using MTT assay (Figure 1c). To elucidate the effect of MM1 on cell proliferation, cells treated with 75 *μ*M of magnolol or MM1 for 24 h were analyzed by flow cytometry using propidium iodide staining, As shown in Figure 1d, the percentage of cells in the sub-G1 region (M1) was greatly increased in the cells treated with MM1 when compared to the cells treated with magnolol or mock control, suggesting that the induction of apoptosis by MM1 accounts for its potent anti-proliferation activity. Taken together, these data showed that the structural features contributing to the greatest cytotoxic effects on skin cancer cells are a hydroxyl with a methoxy moiety, as found in MM1.

### Effects of magnolol and MM1 on the clonogenic and anchorage-independent growth ability of skin cancer cells

**Figure 2:**
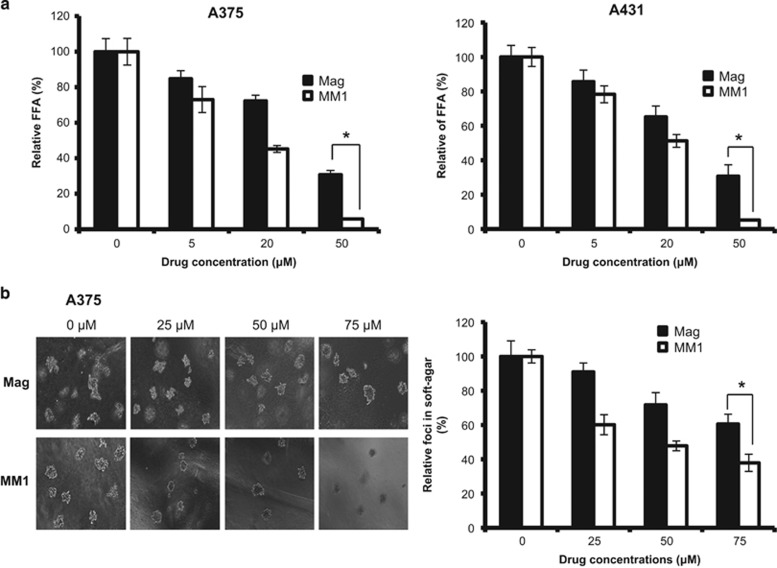
Effects of magnolol and MM1 on the clonogenic ability and anchorage-independent growth of skin cancer cells. (**a**) A375 (left panel) and A431 (right panel) cells were treated with various concentrations of magnolol or MM1 for 6 days and then cultured for an additional 8 days in the absence of drugs. The numbers of foci were scored, and the data are presented as the relative focus-forming ability (FFA). (**b**) A375 cells were treated with different concentrations of magnolol and MM1. The anchorage-independent growth was assessed as described in the Materials and Methods section. Data are expressed as the mean±S.D. of three independent experiments. Symbols: *P<0.05, as analyzed by unpaired *t*-tests

Next, we examined the effects of magnolol and the highly potent compound MM1 on the clonogenic and anchorage-independent growth ability of A375 and A431 cells. As shown in Figure 2a, MM1 displayed significant greater suppression of clonogenic ability than magnolol when treated at 50 *μ*M. Similarly, MM1 displayed a significantly greater suppression of anchorage-independent growth ability than magnolol when treated at 75 *μ*M in A375 cells (Figure 2b). A431 cells are incapable of anchorage-independent growth, and thus were not tested in this set of experiments. These results indicated that MM1 has a significant inhibitory effect than magnolol in the colony formation and anchorage-independent growth of skin cancer cells.

### Effects of magnolol and MM1 on the induction of apoptosis in melanoma cells

**Figure 3:**
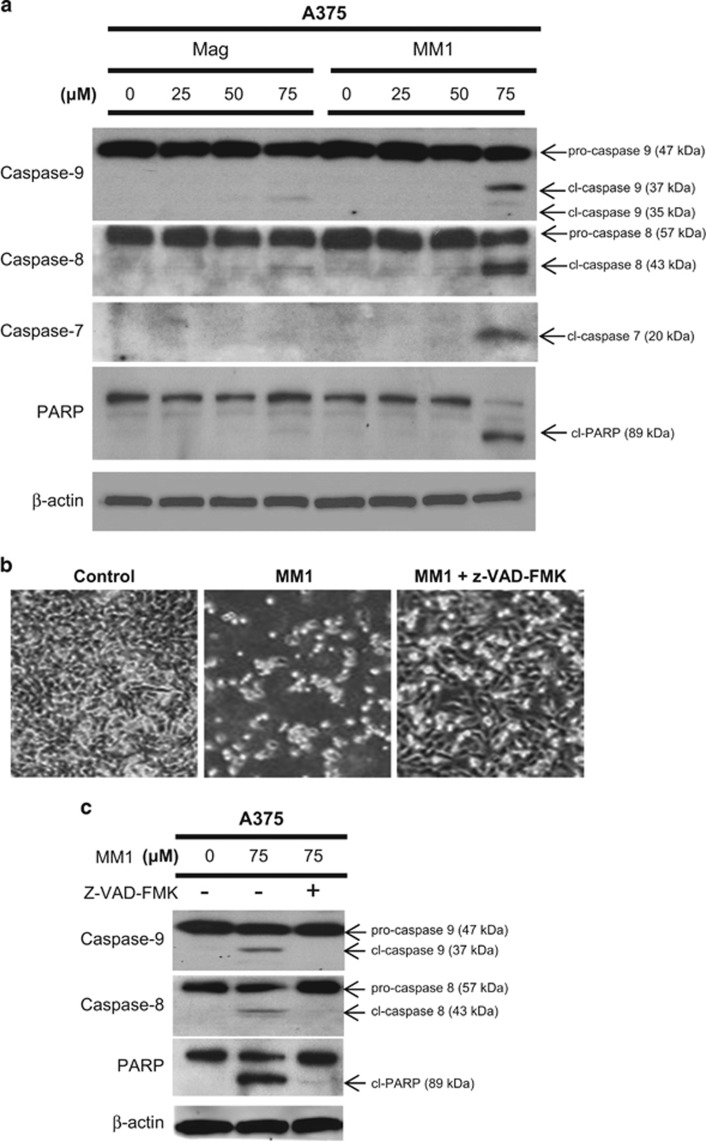
Effect of magnolol and MM1 on the induction of apoptosis in A375 cells. (**a**) A375 cells were treated with magnolol or MM1 for 24 h, and western blotting was used to assess cell lysates for the cleavages of PARP and procaspase-7, -8, and -9. (**b** and **c**) Effect of caspase inhibition on the apoptosis of MM1-treated A375 cells. A375 cells were treated with 75 *μ*M MM1 in the presence or absence of the broad-spectrum caspase inhibitor, z-VAD-fmk (40 *μ*M), for 24 h. Phase-contrast images of MM1-treated cells treated with or without z-VAD-fmk are shown in **b**. Western blot analysis was used to assess cell lysates for the cleavages of procaspase-7, -8, and 9, with *β*-actin used as a loading control (**c**)

To investigate whether MM1-induced cell death is associated with the induction of apoptosis, we analyzed the kinetics of caspase activation in A375 cells. As shown in Figure 3a, the cleavages of caspase-7, caspase-8, caspase-9, and PARP were readily detected as early as 24 h after cells were exposed to 75 *μ*M MM1. Morphological examination using phase-contrast microscopy indicated that while the untreated control cells displayed a normal morphology, the MM1-treated cells displayed a loss of adherence, condensation of cytoplasm, and the formation of apoptotic bodies, which are all indicative of apoptosis (Figure 3b). These results indicate that MM1 displays a greater ability than magnolol to induce apoptosis in A375 cells. Treatment of A375 cells with the broad-spectrum caspase inhibitor, z-VAD-fmk, effectively inhibited the MM1-triggered inductions of apoptotic phenotypes (Figure 3b) and caspase activity (Figure 3c), indicating that apoptosis plays a critical role in the cytotoxic mechanism of MM1.

### Antitumor effects of magnolol and MM1 *in vivo*

**Figure 4:**
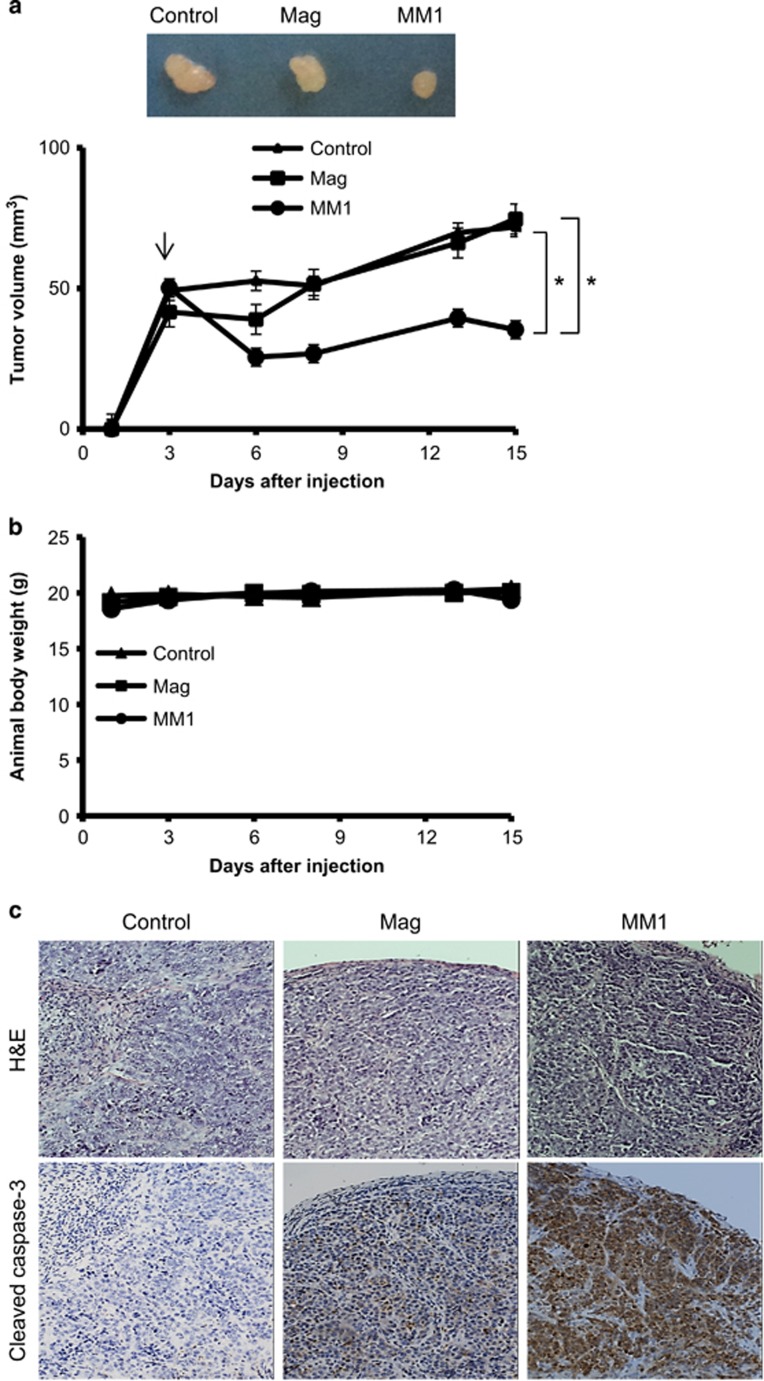
Effects of magnolol and MM1 on the growth of xenograft tumors in nude mice. A375 cells were injected subcutaneously into the flank of each mouse. When the tumor volumes reached about 50 mm^3^, the mice were i.p. injected with magnolol (1 mM in 100 *μ*l acetone), MM1 (1 mM in 100 *μ*l acetone), or DMSO (control) three times per week (*n*=6 per group). The tumor volume (**a**) was determined twice weekly, while body weight (**b**) was measured daily. The xenograft tumors were excised from the mice at the end of experiment in 15 days. The sizes of representative tumors excised from the different groups are shown at the top of **a**, while hematoxylin–eosin staining (magnification, × 200, upper panel) and IHC staining for activated caspase-3 (magnification, × 200, lower panel) are shown in **c**. The results shown in **a** and **b** are presented as the means±S.D. of six mice. Symbols: *P<0.05 by unpaired *t*-tests

The *in vivo* antitumor activities of magnolol and MM1 were evaluated using A375 cell xenografts in nude mice. When the A375 cell xenografts reached about 50 mm^3^, we administered magnolol or MM1 0.1 *μ*mol in 100 *μ*l of acetone by intra-peritoneal (i.p.) injection three times per week. Tumor volumes were measured at the indicated times. As shown in Figure 4a, the i.p. administration of MM1 significantly reduced tumor growth, whereas magnolol had little such effect. Under our experimental conditions, there was no noticeable change in body weight or overt sign of toxicity in the treated mice (Figure 4b). The histological appearance of the excised tumor xenograft by hematoxylin–eosin staining revealed that the entire tumor mass consisted of melanoma cells (Figure 4c; upper panel). Immunohistochemistry (IHC) staining of the excised tumor xenografts revealed no staining of activated caspase-3 in untreated tumor xenografts, whereas weak and heavy staining of activated caspase-3 were detected in magnolol- and MM1-treated tumors, respectively (Figure 4c; lower panel). In agreement with the results of our *in vitro* studies, these results indicate that MM1 displays greater antitumor activity than magnolol against skin cancer cells *in vivo*.

### Transcriptomic and pathway analyses of MM1-treated cells

**Figure 5:**
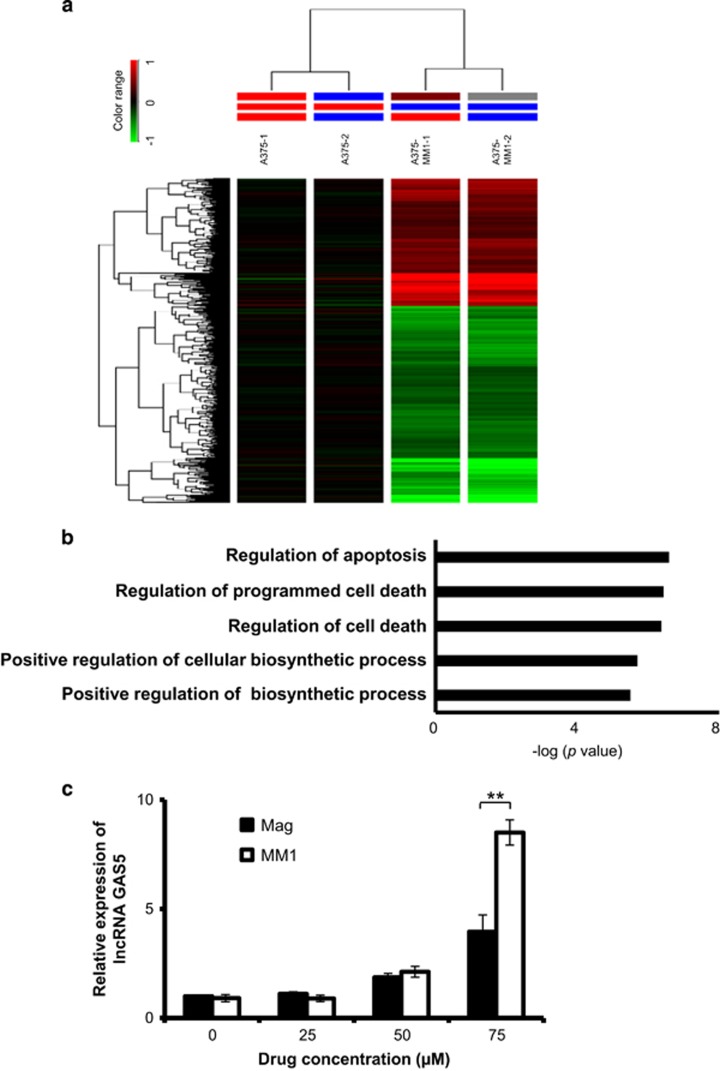
MM1 upregulates lncRNA GAS5. A375 cells were treated with or without 50 *μ*M MM1 for 24 h, and total RNA was subjected to microarray analysis as described in the Materials and Methods section. The heatmap shown in **a** presents a hierarchical clustering of the transcriptome profiles from duplicate cultures of untreated and MM1-treated A375 cells. The relative expression of each RNA transcript is indicated by color, and ranges from red (higher expression) to green (lower expression). (**b**) Functional classification of the differentially expressed genes in MM1-treated A375 cells, as assessed using the DAVID Database web server. The differentially expressed proteins are linked to a number of biological processes. (**c**) Upregulation of lncRNA GAS5 in magnolol- or MM1-treated A375 cells. A375 cells were treated with the indicated concentrations of magnolol or MM1 for 24 h, and the expression levels of lncRNA GAS5 were determined by real time RT-PCR, as described in the Materials and Methods section. The expressions of lncRNA GAS5 in the magnolol- and MM1-treated cells were normalized to that of the untreated cells, and are presented as relative expression levels. The data shown represent the mean±S.D. of three independent experiments. Symbol: **P<0.01, as analyzed with the unpaired *t*-test

To explore the molecular actions of MM1 on A375 cells, we used Affymetrix human GeneChips to compare the gene expression profiles of A375 cells treated with or without MM1. A total of 1270 genes displayed differential expression over 1.5-fold between treated and untreated A375 cells (Figure 5a). We subjected these genes to pathway analyses using the DAVID Database web server, and performed gene ontology prediction. Our results suggested that MM1 could affect certain key factors involved in regulating apoptosis and programmed cell death (Figure 5b). Since recent studies have indicated that long non-coding RNAs (lncRNAs) are also involved in regulating various physiologic functions, including proliferation, migration, and apoptosis,^28, 29, 30, 31, 32^ we examined whether any of the differentially expressed genes represented lncRNAs. We found that the differentially expressed genes included 64 lncRNAs. The 20 putative target lncRNAs with the highest scores are listed in Table 1. They include lncRNA *growth arrest-specific 5* (GAS5), which is a known tumor suppressor that promotes growth arrest and/or apoptosis in multiple cell types.^33, 34^ Overexpression of lncRNA GAS5 was shown to reduce invasion in human melanoma cells,^34^ while knockdown of lncRNA GAS5 was reported to abolish cell cycle arrest at G1 phase in stomach cancer.^35^ We thus selected lncRNA GAS5 for further study. To address the potential importance of lncRNA GAS5 upregulation for the antitumor activity of MM1, we first examined whether MM1 more effectively induced lncRNA GAS5 compared to magnolol. As shown in Figure 5c, quantitative real-time RT-PCR revealed that lncRNA GAS5 was increased to a higher level in MM1-treated cells compared to magnolol-treated cells.

### lncRNA GAS5 plays a critical role in the MM1-mediated induction of apoptosis

**Figure 6:**
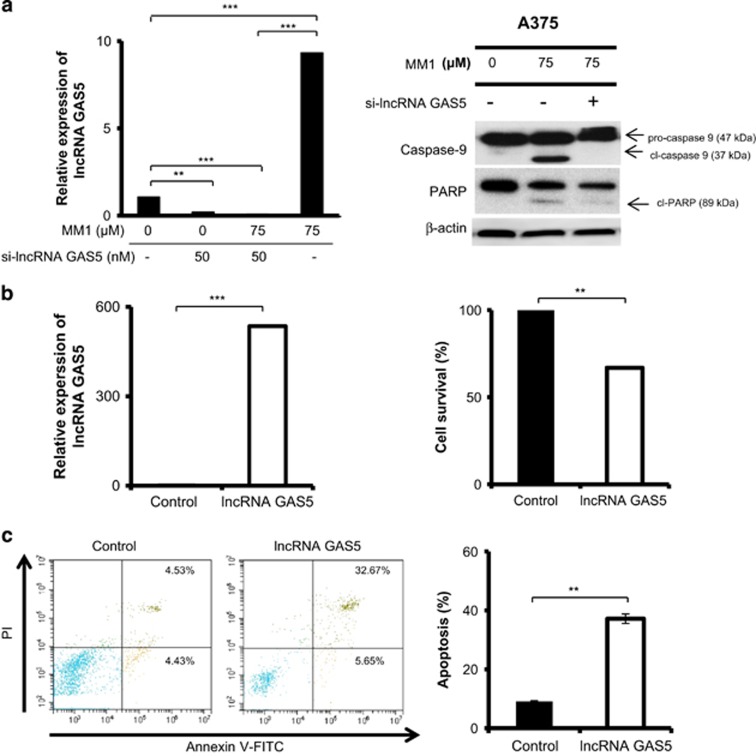
lncRNA GAS5 plays a critical role in the MM1-mediated induction of apoptosis. (**a**) Effect of lncRNA GAS5 depletion on cell apoptosis. Left panel: A375 cells were transfected with siRNA against lncRNA GAS5 (si-lncRNA GAS5) at 50 nM or with nontargeting siRNA(−). After 48 h, the transfected cells were treated with 75 *μ*M of MM1 for 24 h. The levels of lncRNA GAS5 were assessed by real-time RT-PCR and expressed as relative to that of non-MM1-treated control (Left panel). The cleavage of PARP and caspase-9 was analyzed by western blotting (Right panel). *β*-actin served as an internal control. (**b** and **c**) Effect of lncRNA GAS5 overexpression on cell proliferation and apoptosis. A375 cells were transfected with pCDNA3.1-lncRNA GAS5 or the empty vector (control). After 48 h, the transfected cells were assayed for expression of lncRNA GAS5 (**b**, left panel), for viability by MTT method (**b**, right panel), and for the induction of apoptosis by phosphatidylserine exposure with Annexin V-FITC using flow cytometry (**c**). Data are expressed as the mean±S.D. of three independent experiments. Symbols: **P<0.01; and ***P<0.001, as analyzed by unpaired *t*-tests

To test whether lncRNA GAS5 is involved in the MM1-induced apoptosis, A375 cells were transfected with siRNAs targeting lncRNA GAS5 for 48 h and the effect of lncRNA GAS5 depletion on the MM1-induced apoptosis was evaluated. Treatment of A375 cells with siRNAs targeting lncRNA GAS5 effectively reduced the level of lncRNA GAS5 and abolished its induction by MM1 (Figure 6a, left panel). Depletion of lncRNA GAS5 inhibited the ability of MM1 to induce apoptosis (Figure 6a, right panel). To further investigate the role of lncRNA GAS5 in the mediation of skin cancer cell viability and apoptosis, the A375 cells were transfected with pCDNA3.1-lncRNA GAS5 plasmid DNA to evaluate the effect of ectopically expressed lncRNA GAS5 on cell viability and apoptosis. Transfection of lncRNA GAS5-expressing plasmid in A375 cells resulted in an increased expression of lncRNA GAS5 about 500-fold (Figure 6b, left panel), reduced the cell proliferation ~60% (Figure 6b, right panel), and increased the induction of apoptosis by ~4-fold (Figure 6c).

### lncRNA GAS5 is downregulated in skin cancer tissues

**Figure 7:**
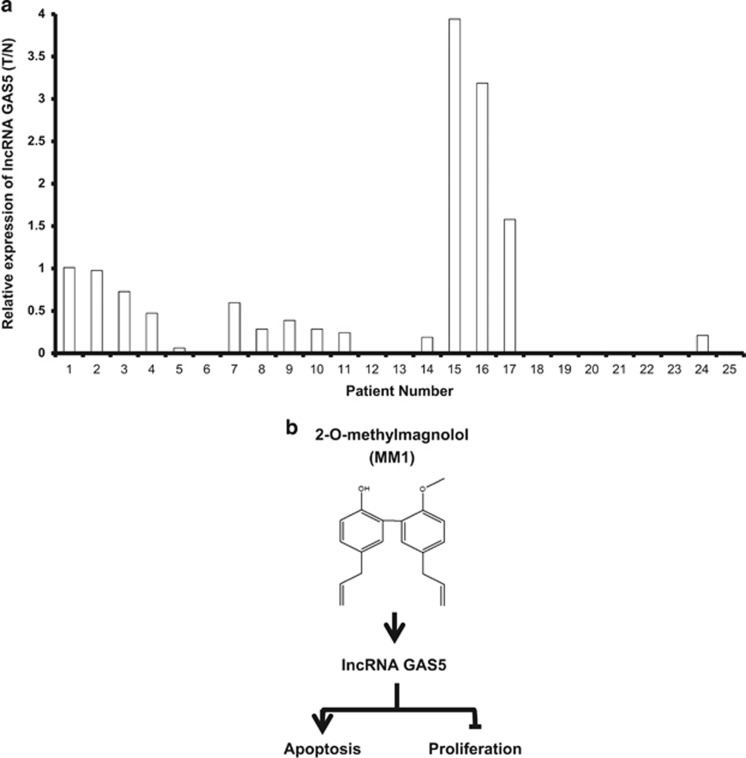
Expression of lncRNA GAS5 in skin cancer patients and model for antitumor activity of MM1. The levels of lncRNA GAS5 in the skin cancer (T) and adjacent normal (N) tissues of each patient were determined by quantitative RT-PCR analysis, normalized with respect to GAPDH, and expressed as the ratio of T/N in the histograms shown in **a**. A schematic representation of the proposed antitumor activity of MM1 is shown in **b**

To explore the possibility that the expression of lncRNA GAS5 may be relatively low in human skin cancer and thus amenable to the treatment by MM1, we employed quantitative RT-PCR to examine the relative expression levels of lncRNA GAS5 in the skin cancer tissues (T) and adjacent normal skin tissues (N) from 25 skin cancer patients. The ratio of relative expression (T/N) was smaller than 0.5 in 18 out of the 25 patients entered in this study (Figure 7a). Therefore, lower expression of lncRNA GAS5 appears to prevail in human skin cancer and strategy of targeting lncRNA GAS5 could be considered as another approach for the treatment of skin cancer.

## Discussion

We previously reported that MM1, a new derivative of magnolol bearing a methoxy moiety, exhibits significantly improved anti-inflammatory activity compared to magnolol, yet has negligible skin toxicity.^27^ Here we report that MM1 also displays better antitumor activity than magnolol against cultured skin cancer cells (Figures 1,2) and in a xenograft animal model *in vivo* (Figure 4). In addition, similar results were also observed in oral cancer cell lines (Supplementary Figure 1). These findings suggest that MM1 could be more therapeutically relevant than magnolol for clinical applications.

Regarding the molecular basis underlying the improved antitumor activity of MM1, we found that MM1 appears to have a greater ability to induce apoptosis (Figure 3) and is predicted to affect certain key factors involved regulating apoptosis and programmed cell death (Figure 5b). These factors include 64 examples of lncRNAs (Table 1), which are now recognized as a major component of the human transcriptome, and have been shown to regulate many key biological processes.^36^ The differentially expressed lncRNAs included GAS5 (upregulated), which is a tumor suppressor lncRNA that promotes growth arrest and/or apoptosis in multiple cell types.^33, 34^ Our real-time RT-PCR assay confirmed that lncRNA GAS5 is indeed upregulated in MM1-treated cells (Figure 5c), suggesting that this lncRNA may contribute to the enhanced apoptosis induction and improved antitumor and anti-metastatic activities of MM1. Indeed, knockdown of lncRNA GAS5 reduced MM1-mediated apoptosis in A375 cells (Figure 6a). In contrast, overexpression of lncRNA GAS5 decreases cell viability and promotes the apoptosis of skin cancer cells (Figures 6b and c). Therefore, MM1-induced upregulation of lncRNA GAS5 can promote apoptosis and inhibit cell proliferation in skin cancer cells (Figure 7b). Our finding is consistent with the known activity of lncRNA GAS5 to induce apoptosis in several cancer cells.^34^ As yet, the molecular basis of lncRNA GAS5-mediated induction of apoptosis remains poorly understood at present. Further investigations on the mechanisms of lncRNA GAS5-induced apoptosis are urgently needed.

In this context, it is interesting to note that the expression of lncRNA GAS5 is relatively low in most of the human skin cancer tissues when compared to their adjacent normal tissues (Figure 7a). Low expression of lncRNA GAS5 was also observed in several other cancers, including breast and lung cancers.^37, 38^ Cancer patients with low expression of lncRNA GAS5 may be amenable to the treatment by MM1. Further test of MM1 for its potential clinical application against human cancers is warranted.

## Materials and methods

### Culture media and antibodies

Culture media and fetal bovine serum were purchased from Life Technologies (Grand Island, NY, USA). Antibodies against cleaved PARP (Asp214) and caspase-7, -8, and -9 were purchased from Cell Signaling (Temecula, CA, USA). The antibody against *β*-actin was purchased from Sigma (St. Louis, MO, USA).

### Compounds

Magnolol was isolated from *Magnolia cortex*,^27^ and MM1 was prepared as described by Lin *et al.*^27^

### Tissues

The human skin cancer and corresponding non-cancerous normal tissues used in this study were obtained from 25 skin cancer patients who underwent surgical resection at Lin-Kou Chang Gung Memorial Hospital. This study was approved by the Ethics Committee of Chang Gung Memorial Hospital, and written informed consent was obtained from each patient.

### Cell culture

A375 (melanoma) and A431 (squamous cell carcinoma) cells were obtained from the American Type Culture Collection (Manassas, VA, USA). SAS, a high-grade tumorigenic human tongue squamous cell carcinoma, was obtained from the Japanese Collection of Research Bioresources (Tokyo, Japan).^39^ The cells were cultured in Dulbecco's modified Eagle's medium containing 10% fetal bovine serum, 0.5 mM sodium pyruvate, 1.2 g/l sodium bicarbonate, and 2.5 mM L-glutamine.

### SRB colorimetric assay for cytotoxicity screening

Cells were seeded in 96-well tissue plates at a density of 4 × 10^4^ cells in 200 *μ*l medium per well. Compounds were added at the indicated concentrations or wells were left untreated as controls, and the plates were incubated for 24 h. Viable cells were detected using the SRB colorimetric method. Briefly, 150 *μ*l 50% trichloroacetic acid (Sigma) was added to each well and the plates were incubated at 4 °C for 1 h. The plates were then washed five times with ddH_2_O, and then air-dried. SRB solution (50 *μ*l/well; Sigma) was added, and the plates were incubated for 30 min at room temperature. The plates were washed five times with 1% acetic acid, air dried, and treated with 10 mM Tris base (pH 10). Absorbance was measured using a microplate reader at 565 nm.

### Assays for cell proliferation, clonogenic ability, and anchorage-independent growth

Assays for cell proliferation (MTT), clonogenic ability, and anchorage-independent growth were performed as described previously.

### Flow cytometric analysis of cell cycle

Cells were fixed in −20 °C absolute ethanol for 4 h and resuspended in 1 ml of PBS containing 20 *μ*g/ml DNase-free, RNaseA. After incubating at 37 ^o^C for 30 min, the cells were treated with propidium iodide (100 *μ*g/ml) at room temperature for 10 min in the dark. A total of 10,000 cells were analyzed by flow cytometry (BD FACSCalibur TM system, Becton–Dickinson, Franklin Lakes, NJ, USA).

### DNA microarray analysis

RNA extraction was performed as previously described.^40^ Total RNA (0.2 *μ*g) was amplified using an Agilent Low Input Quick-Amp Labeling kit (Agilent Technologies, Santa Clara, CA, USA) and labeled with Cy3 (CyDye, Agilent Technologies) during the *in vitro* transcription process. Cy3-labled cRNA (0.6 *μ*g) was fragmented to an average size of about 50–100 nucleotides by incubation with fragmentation buffer at 60 °C for 30 min. Correspondingly fragmented labeled cRNA was pooled and hybridized to Agilent SurePrint G3 Human Gene Exp V3 arrays (Agilent Technologies) at 65 °C for 17 h. Hybridized microarrays were washed, dried under nitrogen, and scanned with an Agilent microarray scanner (Agilent Technologies) at 535 nm for Cy3. Scanned images were analyzed using the Feature extraction10.5.1.1 software (Agilent Technologies). Image analysis and normalization software were used to quantify the signal and background intensity for each feature. The raw microarray data were uploaded to the DAVID Database (http://david.abcc.ncifcrf.gov/) for pathway analysis.

### Western blotting

Western blotting was performed as described previously.^39^

### Detection of lncRNA GAS5 by quantitative real time

RNA from skin cancer cells was extracted as previously described.^40^ RNA from each tissue was isolated using an RNeasy mini kit (QIAGEN, Gaithersburg, MD, USA), followed by treatment with RQ1 RNase-free DNase (Promega, Madison, WI, USA) according to the manufacturer's instructions. Two micrograms of treated RNA sample was subjected to reverse transcription (RT).^41^ lncRNA GAS5 was detected by quantitative real time RT-PCR using the TaqMan non-coding RNA expression assay (product number Hs03464472 m1; Applied Biosystems) as described previously.^42^ GAPDH was used as an internal control.

### RNA interference (RNAi)

Target genes were downregulated by RNAi-mediated inhibition of RNA expression, using a mixture of four siRNAs for each target gene (ON-TARGETplus SMARTpool; Dharmacon, Lafayette, CO, USA) as previously described.^40^ The siGENOME nontargeting siRNA pool (Dharmacon) was used as the control. The selected siRNA sequences were submitted to BLAST searches to ensure that each siRNA targeted only one human gene. The four siRNAs targeting human lncRNA GAS5 (GenBank accession no. NR_002578.2) covered the following: nucleotides 385–403 from the start codon (lncRNA GAS5-1: 5′-AGGCAGACCUGUUAUCCUA-3′), nucleotides 248–266 (lncRNA GAS5-2: 5′-UGGAUGACUUGCUUGGGUA-3′), nucleotides 567–585 (lncRNA GAS5-3: 5′-GAUGGAGUCUCAUGGCACA-3′), and nucleotides 301–319 (lncRNA GAS5-4: 5′-AGGUAUGGAGAGUCGGCUU-3′). In brief, exponentially growing cells were seeded in regular growth medium without antibiotics at 40–50% confluence. After 24 h, cells were transfected with siRNA using the Dharmafect 1 transfection reagent (Dharmacon) according to the manufacturer's instructions. The cells were then incubated for an additional 48 h before experiments.

### Plasmid and transfection

pCDNA3.1-lncRNA GAS5, a CMV-based expression and neomycin-selective plasmid containing lncRNA GAS5, was constructed by GenScript Co. (Piscataway, NJ, USA). Transfection of plasmid DNA into cells was performed using Lipofectamine 2000 (Invitrogen, Carlsbad, CA, USA) according to the manufacturer's protocol.

### Annexin V-FITC and propidium iodide staining assay

A375 overexpressed with pCDNA3.1-lncRNA GAS5 or vector control was plated in 6-well plates. After 48 h incubation, the cells were collected and subjected to Annexin V and propidium iodide staining by using Vybrant Apoptosis Assay Kit 2 (Invitrogen) according to the manufacturer's protocol. After staining, flow cytometry was performed to quantify apoptotic cells.

### Assay for antitumor activity *in vivo*

A xenograft mouse model was used to examine the antitumor activity of the tested compounds against human skin cancer cells. Six-week-old male BALB/c nu/nu mice (National Laboratory Animal Center, Taipei City, Taiwan) were maintained in an SPF (specific pathogen free) environment. Animals (*n*=6 per group) were inoculated subcutaneously in the right flank with A375 tumor cells (2 × 10^6^) in a volume of 100 *μ*l on day 0. Treatment was initiated when the tumors were 50 mm^3^. Mice were randomized to three groups: treatment with magnolol, MM1, or vehicle (control). The tested agents were administered by i.p. injection three times per week. Mice were randomly divided into three groups when the tumors were 50 mm^3^ and treated with acetone as control, magnolol, or MM1 0.1 *μ*mol in 100 *μ*l of acetone by i.p. injection three times per week. Tumor width (W) and length (L) were measured once a week with a caliper, and the tumor volume (V) was calculated according to the formula: V=0.5 × W^2^ × L. All animal experiments were performed in accordance with the guidelines for the Animal Care Ethics Commission of Chang Gung University under an approved animal protocol.^42^

### IHC and hematoxylin–eosin staining

IHC and hematoxylin–eosin staining were performed as described previously.^42^ The primary antibody used for IHC staining targeted activated cleaved-caspase 3 (9546; Cell Signaling).

### Statistics

Statistical differences were evaluated using the Student's *t*-test, and were considered significant at *P*<0.05. The presented results are representative of three independent experiments with similar results.

## Figures and Tables

**Table 1:** List of the 20 highest-scored lncRNA differentially expressed in MM1-treated A375 cells

	*Gene symbol*	*Fold change*	*Description*	*GenBank accession*
1	PITRM1-AS1	5.34	Homo sapiens PITRM1 antisense RNA 1 (PITRM1-AS1)	NR_038284
2	LOC100130987	3.79	Homo sapiens uncharacterized LOC100130987 (LOC100130987)	NR_024469
3	LINC00520	2.63	Homo sapiens long intergenic non-protein coding RNA 520 (LINC00520),	NR_026796
4	LOC728730	2.30	Homo sapiens uncharacterized LOC728730 (LOC728730)	NR_037875
5	MCF2L-AS1	2.23	Homo sapiens MCF2L antisense RNA 1 (MCF2L-AS1)	NR_034002
6	LINC01291	2.23	Homo sapiens long intergenic non-protein coding RNA 1291 (LINC01291)	NR_125792
7	CECR5-AS1	2.09	Homo sapiens CECR5 antisense RNA 1 (CECR5-AS1)	NR_024482
8	SLCO4A1-AS1	2.03	Homo sapiens SLCO4A1 antisense RNA 1 (SLCO4A1-AS1)	NR_024470
9	LINC01023	2.01	Homo sapiens long intergenic non-protein coding RNA 1023 (LINC01023)	NR_046368
10	PCAT6	1.98	Homo sapiens prostate cancer associated transcript 6 (non-protein coding) (PCAT6)	NR_046325
11	LOC101929715	1.93	Homo sapiens uncharacterized LOC101929715 (LOC101929715)	NR_110597
12	NEAT1	1.89	Homo sapiens nuclear paraspeckle assembly transcript 1 (non-protein coding) (NEAT1)	NR_028272
13	LINC01296	1.87	Homo sapiens long intergenic non-protein coding RNA 1296 (LINC01296)	NR_122112
14	LOC100133669	1.83	Homo sapiens uncharacterized LOC100133669 (LOC100133669)	NR_026913
15	LINC00632	1.80	Homo sapiens long intergenic non-protein coding RNA 632 (LINC00632)	NR_104228
16	GAS5	1.78	Homo sapiens growth arrest-specific 5 (non-protein coding) (GAS5)	NR_002578
17	FLJ46906	1.77	Homo sapiens uncharacterized LOC441172 (FLJ46906)	NR_033896
18	MALAT1	1.76	Homo sapiens metastasis associated lung adenocarcinoma transcript 1 (non-protein coding) (MALAT1)	NR_002819
19	LOC101929596	1.75	Homo sapiens uncharacterized LOC101929596 (LOC101929596)	NR_110259
20	LOC100131564	1.72	Homo sapiens uncharacterized LOC100131564 (LOC100131564)	NR_034089
