# Supplementary figures and images for: 2-O-Methylmagnolol upregulates the long non-coding RNA, GAS5, and enhances apoptosis in skin cancer cells
Source: Cell Death Dis. 2017 Mar 2;8(3):e2638–. doi: 10.1038/cddis.2017.66 (PMC5386561; doi:10.1038/cddis.2017.66)

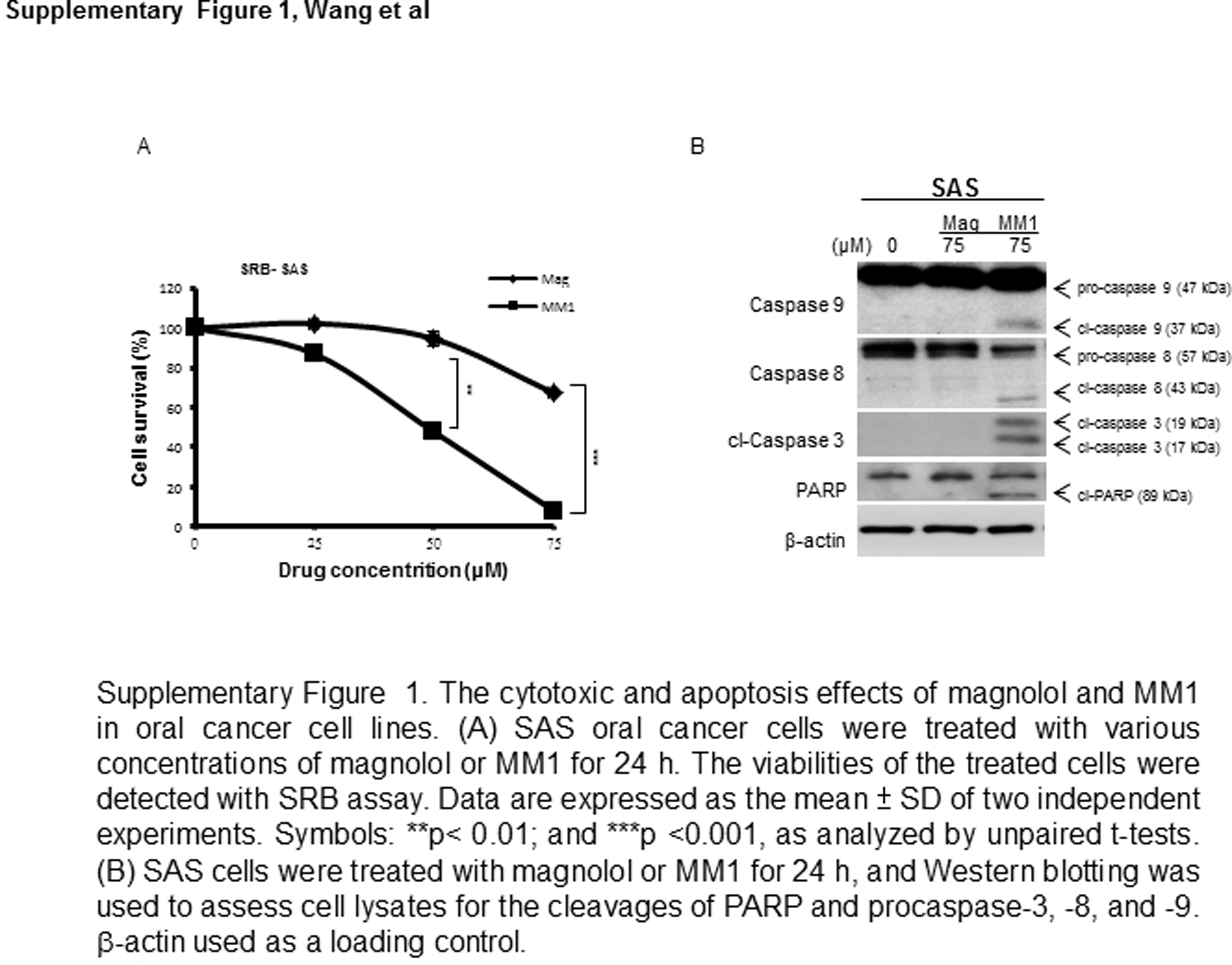

Supplement: Supplementary Information [file cddis201766x1.tif]
